# Anticancer Activity of *Ipomoea purpurea* Leaves Extracts in Monolayer and Three-Dimensional Cell Culture

**DOI:** 10.1155/2021/6666567

**Published:** 2021-06-07

**Authors:** F. Beheshti, A. A. Shabani, M. R. Akbari Eidgahi, P. Kookhaei, M. Vazirian, M. Safavi

**Affiliations:** ^1^Research Center of Biotechnology, Semnan University of Medical Sciences, Semnan 009823, Iran; ^2^Department of Biotechnology, School of Medicine, Semnan University of Medical Sciences, Semnan 009823, Iran; ^3^Cancer Research Center, Semnan University of Medical Sciences, Semnan 009823, Iran; ^4^Department of Pharmacognosy, Faculty of Pharmacy, Tehran University of Medical Sciences, Tehran 009821, Iran; ^5^Department of Biotechnology, Iranian Research Organization for Science and Technology, 13353-5111, Tehran 009821, Iran

## Abstract

Cancer is a leading cause of death and a vital health care challenge in the world. Hence, this work was conducted to determine the *in vitro* anticancer property and also the molecular mechanism of aqueous and organic extracts of *Ipomoea purpurea* leaves in three human cancer cell lines, including A-549 (human lung cancer), HepG-2 (human liver cancer), MDA-MB-231 (human breast cancer), and MCF-10A (breast normal cell line). *In vitro* cytotoxic potential of organic extracts, such as hexane, chloroform, ethyl-acetate, methanol, and aqueous extract was examined using a standard (3-(4,5-dimethylthiazole)-2,5-diphenyltetrazolium bromide) MTT method in both monolayer two-dimensional (2D) and spheroids multicellular three-dimensional (3D) cultures. The MTT assay data showed that methanol and chloroform extracts of *I. purpurea* leaves had the antiproliferative effect on lung and breast cancer cells with IC_50_ of 53.62 ± 0.07 and 124.5 ± 0.01 *µ*g/mL, respectively. The results of further examinations, such as dual acridine orange/ethidium bromide, Annexin V-FITC/PI, and caspase-3 colorimetric assay, confirmed that methanol and chloroform extracts of *I. purpurea* as the most potent cytotoxic extracts might contain a variety of phytochemicals, promoting apoptosis in lung and breast cancer cells. The present research findings suggested that methanolic extract of *I. purpurea* leaves induced S-phase cell cycle arrest and intrinsic pathway of apoptosis in A-549 lung cancer cells. The study further showed that *I. purpurea* could be a helpful candidate for cancer treatment.

## 1. Introduction

Cancer is one of the most vital health care challenges worldwide [1, 2]. Its prevalence and corresponding death indicate its growth rate in developing and developed countries [3, 4]. The cancer incidence is on a stable increase rate, with 7.6 million deaths in 2008, which has been predicted to double in 2030 [2]. Despite signiﬁcant investment and advances in cancer chemotherapy, the patient's survival has not improved significantly in many countries [5]. According to numerous reports, there is no anticancer treatment with 100% efficacy and no side effects [2–4]. Hence, there remain an essential need for new drug discovery resulting in capable anticancer agents to defeat the problems related to chemotherapeutics, such as drug resistance and toxicity [3]. Due to the severe adverse effects of current chemotherapeutics, much attention is being paid to plants as natural sources to ameliorate cancer treatment [6, 7]. More than 60% of approved anticancer drugs are either natural products or their derivatives; thus, herbs serve as a significant source of anticancer agents [8–11]. As of now, over 3,000 species of plants with anticancer properties have been recognized [12]. The plant materials are used broadly in the world due to better cultural acceptability, fewer adverse effects, and compatibility with the human body [6, 13]. Traditionally, medicinal herbs have been commonly used in treating various human diseases. This use is usually due to the presence of phytochemicals, known as plant metabolites [14]. According to some research works, the anticancer activity of plants is associated with a range of natural compounds, such as catechins, polyphenols, and ﬂavonoids [7, 12, 13]. The genus *Ipomoea*, with more than 500 species, belongs to the Convolvulaceae family consisted of nearly 1,650 predominantly tropical species. From ancient times, some important species of *Ipomoea* have been extensively used for different medicinal and nutritional purposes [15].

The flowers of *Ipomoea purpurea* contain cyanidins and pelargonidins, which have antioxidant properties [16]. This species is also known for entheogen activities together with *tricolor* and *violacea*, which are rich in ergoline derivatives, isoergine, ergine, and lysergol [17]. For the first time, the *in vitro* antiproliferative and apoptosis-inducing activities of *I. purpurea* were investigated. Among various strategies used in chemotherapy, one of the most authoritative approaches to developing novel anticancer drugs is the induction of apoptosis. In cancer pathogenesis, apoptosis has a key role, and resistance to this process is one of the most significant indications of human cancer. Among the different forms of cell death, the main controlled type in normal and cancerous cells is caspase-dependent apoptosis. It involves induction and activation of caspases, a family of cysteine proteases, including initiator and effector. Caspase-3 as the main effector and caspase-8 and caspase-9 as central initiator caspases are the critical enzymes in the apoptosis progression [3].

This research aims to determine the anticancer activity of the extracts of *I. purpurea* leaves against a panel of human cancer cell lines of A-549, HepG-2, and MDA-MB-231, besides MCF-10A as a normal cell line. Since apoptosis is considered a significant type of cell death caused by various stimuli, such as medicine, therefore, in this study, the ability of the most potent cytotoxic extracts of *I. purpurea* leaves to induce apoptosis in the human cancer cell lines was investigated. In this regard, the current research was the first paper that elucidated the molecular mechanism of *I. purpurea* leaves extracts in inducing apoptosis in lung and breast cancer lines. The apoptosis-related pathway in the exposed cells was determined by caspase-3 activity assay, procaspase-8 cleavage as the indicator of the extrinsic pathway, and procaspase-9 cleavage as an indicator of the intrinsic way.

## 2. Materials and Methods

### 2.1. Sample Collection and Authentication

The green and fresh leaves of *I. purpurea* were collected from Iran on the campus of the Iranian Research Organization for Science and Technology in September 2018. Then, this plant was recognized and approved by the Botany Department of the Pharmacy Faculty, Tehran University of Medical Sciences. A voucher specimen was deposited into the herbarium under collection number 6595-TEH.

### 2.2. Plant Material and Extraction

The leaves of *I. purpurea* were washed with water and cut into small pieces; then, they were shade-dried, ground to powder using an electric blender, and stored at room temperature before extraction. Two hundred grams of the powdered material was sequentially extracted by solvents of diverse polarities, including hexane, chloroform, ethyl-acetate, methanol, and distilled water. Extraction was done by mechanical stirring at ambient temperature for 24 h (25°C) except for the hot water one (80°C) performed in 30 min. The sample in organic solvents was then filtered using a Buchner funnel and Whatman No. 1 filter paper; then, the residue was extracted more than two times, and supernatants of each solvent were mixed. The aqueous extracts were then concentrated using freeze-drying and the organic extracts by rotary evaporator under vacuum (Heidolph, Heizbad Hei-VAP, Germany) at 20°C. All of these extracts were stored at −20°C for further experiments.

### 2.3. Materials

Fetal bovine serum (FBS), penicillin-streptomycin, and the cell culture medium (RPMI 1640) were prepared from Gibco BRL, UK. The culture plates were provided by SPL (Life Sciences, Korea). Annexin V-FITC/PI kit and PI were obtained from BD Pharmingen™. Ethidium bromide, acridine orange, 3-(4,5-dimethylthiazole)-2,5-diphenyltetrazolium bromide (MTT), and Ac-DEVD-p-nitroaniline (pNA) as a chromogenic substrate of caspase-3 were prepared from Sigma-Aldrich. Caspase-9 and caspase-8 antibodies were provided by Abcam, and mouse anti-*β*-actin antibody was obtained from cell signaling. The secondary antirabbit and antimouse HRP-conjugate antibodies were prepared from Razi BioTech (Tehran, Iran). All other chemicals were provided by Merck (Darmstadt, Germany) and Sigma-Aldrich.

### 2.4. Cell Culture

A panel of different human cancer cell lines, including A-549 (human lung cancer), HepG-2 (human liver cancer), and MDA-MB-231 (human breast cancer), besides one normal cell line (MCF-10A), were provided by the National Cell Bank of Iran. The cells were incubated at 37°C in a humidified atmosphere of 5% CO_2_ in 95% air all over the experiments. RPMI 1640 medium supplemented with 10% heat-inactivated fetal calf serum, penicillin (100 U/mL), and streptomycin (100 *μ*g/mL) was used for typical subculturing and all tests. The trypan blue exclusion test was used to calculate the number of viable cells.

### 2.5. *In Vitro* Cell Viability Assay

The antiproliferation of *I. purpurea* extracts on human cells was screened by the MTT colorimetric method. Briefly, the cells (1.5 × 10^4^ cells/well) were seeded in 96-well plates at 37°C and allowed to attach to the substrate in a humidified atmosphere of 5% CO_2_ and 95% air overnight. After 24 h compatibility of the cells with new conditions, different concentrations of extract (50–400 *µ*g/mL) were added in the triplicate wells and incubated for 24, 48, and 72 h time points. Etoposide and dimethyl sulfoxide (DMSO) with a final concentration of 0.1% were used as positive and negative controls, respectively. After the specific times of exposure to extracts, cells were incubated with a final concentration of 0.5 mg/mL MTT solution. After 4 h incubation, the culture medium was replaced with 100 *µ*L of DMSO to dissolve the formazan crystals. The absorbance was read at 570 nm using a multiwell plate reader (Gen5, Epoch, BioTek). The percentage of cell viability was determined. The concentration of the extract caused a 50% reduction in cancer cell proliferation was considered as IC_50_, which was estimated with the concentration-response curves by nonlinear regression analysis [18].

### 2.6. Spheroid Preparation Using Hanging Drop and Liquid Overlay Methods

Three-dimensional (3D) cultures with many features that mimic *in vivo* microenvironments were used in cellular responses to drug treatments to make a better-informed decision about promising extracts. The 3D multicellular spheroids were formed using a combination of hanging drop and liquid overlay cultivation methods [19].

#### 2.6.1. Hanging Drop

The hanging drop technique was applied to A-549 and MDA-MB-231 cell spheroids generation. Fifty microliter of single-cell suspension of the cells (3 × 10^3^ cells/drop) in complete culture medium was placed on the inner side of a Petri plate lid. The Petri plate was captured upside down on top of the plate after filling the Petri with 3 mL of sterilized phosphate-buffered saline (PBS) to humidify the culture chamber and then incubated for 4 days in a 90% humidified incubator at 37°C with 5% CO_2_. Cells under the force of gravity could be settled and concentrated at the bottom of the drops.

#### 2.6.2. Liquid Overlay

The liquid overlay cultivation technique was used to increase the size of spheroids for extract treatment. After 4 days of incubation in the hanging drop method, the small 4-day-old spheroids were transferred to a new 96-well U-bottom plate precoated with 50 *μ*l of 0.5% poly-HEMA in 95% ethanol and air-dried for 3 days at 37°C before use. The plates were kept under standard cell culture conditions (37°C, 5% CO_2_, and 95% humidity) for at least 3 days. A-549 and MDA-MB-231 cell spheroids were then exposed to various concentrations ranged between 100 and 800 *μ*g/mL of methanol and chloroform extracts, respectively. After incubation periods of 24, 48, and 72 h of the spheroids with extracts, the treated spheroids were transferred to a new 96-well flat-bottom plate. Afterward, the supernatant was removed, 200 *μ*l/well of phenol red-free medium containing MTT solution (0.5 mg/mL) was added, and then the plate was kept for an additional 4 h at 37°C. After forming the blue formazan crystals, the supernatants of each well were replaced with DMSO to dissolve the crystals following spheroids incubation on a shaker at 37°C. Lastly, absorption was read at a wavelength of 570 nm with an ELISA multiwell plate reader (Gen5, Epoch, BioTek).

### 2.7. Dual Acridine Orange/ethidium Bromide Staining Assay

Dual acridine orange/ethidium bromide double-staining technique was widely used to detect morphological changes in cells during the apoptosis process. A-549 and MDA-MB-231cells were developed in 6-well plates (3 × 10^5^ cells/well) and treated with and without IC_50_ values of methanol and chloroform leaves extracts for 24 h. After washing the cells with PBS, cell suspension (9 *µ*L) was transferred to glass slides, stained with 1 *µ*L of dye mixture (100 mg/mL acridine orange and 100 mg/mL ethidium bromide in PBS), then covered with a clean coverslip, and immediately inspected by fluorescence microscope (Axoscope 2 plus, Zeiss). The dual acridine orange/ethidium bromide staining method was repeated three times. Acridine orange, a vital dye, penetrates living and dead cells, emitting green ﬂuorescence by intercalation into DNA. Ethidium bromide is resistant to cells with an intact plasma membrane and emits red ﬂuorescence after intercalation into the DNA of the affected cells. Therefore, it recognizes the population of necrotic and the late stage of apoptotic cells.

### 2.8. Flow Cytometric Analysis of the Apoptotic Cells with Annexin V-FITC/PI Assay

This assay was used to determine the abundance of viable, early apoptotic, late apoptotic, and necrotic cells within a population treated with extracts. Apoptosis was quantified by measuring exposure of phosphatidylserine on the extracellular face of the plasma membrane of the apoptotic cells using an Annexin V-FITC/PI apoptosis detection kit based on the manufacturer's instructions. This assay allows the discrimination of live cells (unstained cells) from Annexin V-FITC-stained apoptotic cells and PI-stained necrotic cells. Briefly, the A-549 and MDA-MB-231 cells were seeded into 6-well plates and incubated overnight before exposure to IC_50_ values of methanol and chloroform leaves extracts and etoposide, as control commercial drug. The cells were harvested, and cell pellets were washed twice with cold PBS after 24 h incubation and resuspended in the binding buffer. Eventually, after resuspending the cells in 5 *μ*l of Annexin V-FITC and 5 *μ*l of the PI solution, the cell suspension was gently mixed, and tubes were incubated in the dark for 15 min at room temperature before analysis (Partec PAS, Germany).

### 2.9. Cell Cycle Analysis

Cell cycle phase distribution was determined by analytical DNA flow cytometry. To perform, flow cytometric measurements of DNA content were identified with 70% ethanol-fixed A-549 cells using PI and UV light. A-549 cells were seeded in 6-well culture plates (3 × 10^5^ cells/well) and incubated overnight. The cells were then exposed to the IC_50_ value of the methanol extract for 24 h. After harvesting, the cells were washed twice with PBS. They were ﬁxed in 70% ethanol at 4°C for 12 h in the dark; after centrifuging, they were again incubated for 30 min at 37°C in a PBS solution containing 1 mg/mL RNase A. PI was added for DNA content analysis. The analysis was performed using a flow cytometer (Partec PAS, Germany), and the percentage of cells in *G*_0_/*G*_1_, S, and *G*_2_/M phases was determined.

### 2.10. Caspase-3 Activation Assay

The activity of caspase-3 was detected 12 and 24 h after treatment of the cells with the extracts through the caspase-3 colorimetric method. In summary, A-549 and MDA-MB-231 cells (3 × 10^5^ cells/well) in 6-well plates were exposed to the IC_50_ values of methanol and chloroform extracts of *I. purpurea* leaves for 12 and 24 h, respectively. Afterward, the control and treated cells were harvested and washed with PBS; then, they were incubated on ice for 10 min in lysis buffer containing 20 mM PIPES (pH 7.4), 2 mM MgCl_2_, 10 mM KCl, 2 mM EDTA, 1 mM EGTA, and 4 mM DTT and freshly were supplemented with protease inhibitor cocktail. The cytosol fraction was transferred to fresh tubes on ice after centrifugation at 10,000 g for 15 min. Protein concentrations were calculated by the bovine serum albumin (BSA) calibration curve using the Bradford method. For each assay, 100 *µ*g protein was incubated with 200 mM colorimetric caspase substrate (Ac-DEVD-pNA) in 100 *µ*L reaction buffer (50 mM HEPES, 0.1% CHAPS, 100 mM NaCl, 10 mM DTT, 0.1 mM EDTA, and 10% Glycerol). The proteolytic cleavage of the labeled Ac-DEVD-pNA by the activated caspase-3 leads to the release of the colored chromophore pNA. In this work, spectrophotometric detection of free molecule pNA was quantitated with the microtiter plate reader. The absorption amount was read at various times using a plate reader (Gen5, Epoch, BioTek) at 405 nm.

### 2.11. Western Blotting Assay

This analysis was performed according to the conventional assay. A-549 cells were cultured in 6-well plates and exposed to the IC_50_ values of methanol and chloroform extracts for 24 h. After incubation, the cells were collected, harvested, and lysed in lysis buffer (20 mM PIPES (pH 7.4), 10 mM KCl, 2 mM MgCl_2_, 1 mM EGTA, 2 mM EDTA, and 4 mM DTT) in the presence of protease inhibitors. The cells were suspended in cold cell lysis buffers, disrupted by being forced (20 times)through syringe aspiration in lysis buffer, and centrifuged at 15,000×g for 30 min at 4°C. After determining protein concentration, the equal amounts of proteins were heated at 95°C, separated through 15% sodium dodecyl sulfate-polyacrylamide gel electrophoresis (SDS-PAGE), and then transferred to the polyvinylidene fluoride (PVDF) membrane. The membrane was blocked in TBST as blocking solution (150 mmol/L NaCl, 50 mmol/L Tris-Cl, pH 7.6, and 0.1% Tween 20) containing 1% (w/v) casein for 2 h. Later, the membrane was incubated with mouse anti-caspase-9 and rabbit anti-caspase-8 primary antibodies overnight, followed by HRP-conjugated antimouse and antirabbit IgG for 2 h. Finally, the blots were developed with ECL advance western blotting detection kit (Amersham).

### 2.12. Statistical Analysis

Experimental data were analyzed using Microsoft Excel 2019 software. Conversely, all measurement values were stated in at least three independent experiments as the mean and standard deviation (SD). The significant differences between means were detected by the Student's *t*-test and one-way analysis of ANOVA with a statistical significance of *P* < 0.01 and *P* < 0.05.

## 3. Results

### 3.1. Antiproliferative Activity

**Figure 1:**
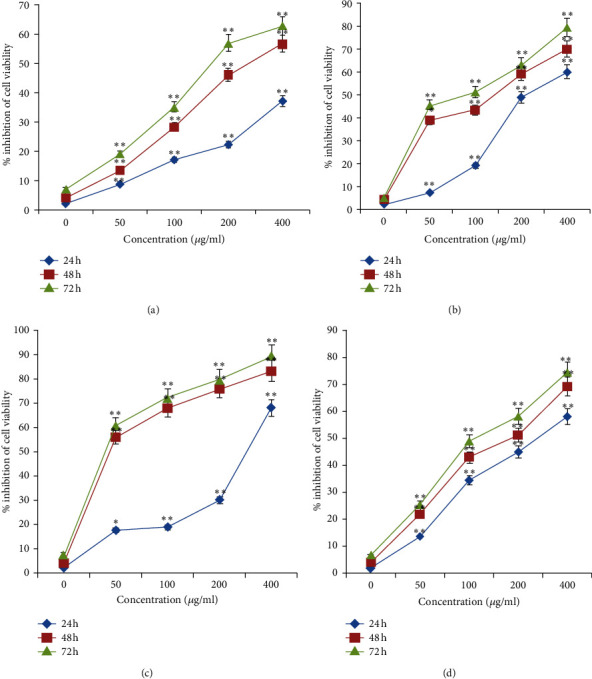
The cell viability inhibition of MDA-MB-231 and A-549 cell lines treated with extracts of *I. purpurea* leaves for 24, 48, and 72 h using the MTT assay. The cell viability inhibition of MDA-MB-231 cell line treated with methanol (a) and chloroform (b) and A-549 cell line treated with methanol (c), and chloroform (d) leaves extracts of *I. purpurea* at the indicated concentrations for 24, 48, and 72 h was determined using the MTT assay. The data are expressed as the mean ± SD of three independent experiments. Significant differences are indicated by ^*∗*^*P* < 0.05 and ^*∗∗*^*P* < 0.01 as compared with the untreated control.

In the current research, the inhibitory activities of aqueous and organic extracts of *I. purpurea* leaves on A-549 (lung cancer), HepG-2 (liver cancer), MDA-MB-231 (breast cancer) human cancer cell lines, and MCF-10A as a normal cell line were examined with the MTT assay. As shown in Tables 1–3 , the methanol and chloroform leaves extracts of *I. purpurea* were the most potent cytotoxic extracts against the A-549 and MDA-MB-23 cancer cell lines, respectively; moreover, the ethyl-acetate extract has cytotoxic effects against A-549 and MDA-MB-23, while the aqueous and hexane extracts of *I. purpurea* leaves did not exhibit anticancer activity against any of the cell lines. Treatment with methanol and chloroform leaves extracts of *I. purpurea* diminished the viability of A-549 and MDA-MB-23 cells in a concentration and time-dependent approach, after 24, 48, and 72 h of exposure (Figure 1). The data revealed that the increase in extracts concentration of up to 400 *μ*g/mL could decrease the cell viability primarily (*P* < 0.05) in a concentration-dependent mode in both cell lines. Conversely, the cell viability inhibition of all cancer cells three times (24, 48, and 72 h) was presented in Supplementary Information (Supplementary Materials). Besides, aqueous and organic extracts were tested in MCF-10A normal cell line to survey their toxicity effects on normal cells. Our present results provided that all extracts had no toxicity on MCF-10A normal cell line till 5,000 *μ*g/mL (Tables 1–3).

**Figure 2:**
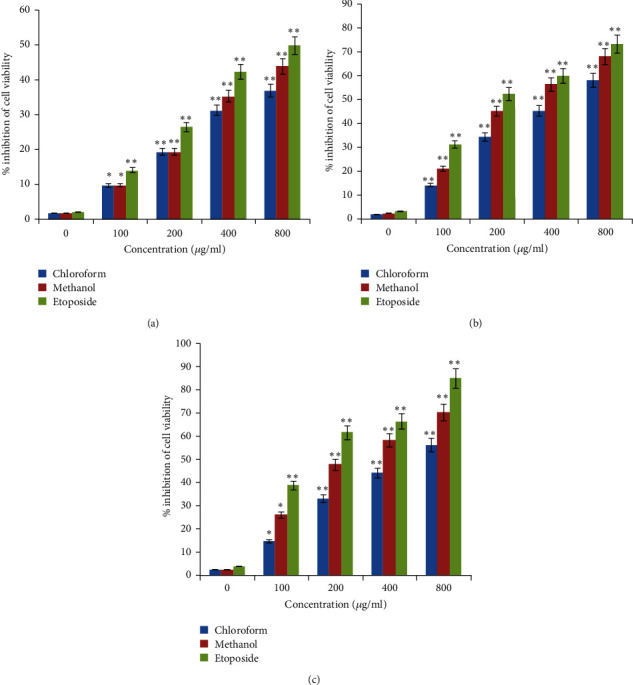
The Inhibition effects of chloroform and methanol extracts on 3D spheroids of MDA-MB-231 and A-549, respectively. Spheroids of MDA-MB-231 and A-549 were treated with different concentrations of chloroform and methanol extracts of *I. purpurea* and etoposide for 24 h (a), 48 h, (b) and 72 h (c). The data shown are the mean ± SD from three independent experiments, each with triplicate wells. Significant differences are indicated by ^*∗*^*P* < 0.05 and ^*∗∗*^*P* < 0.01 as compared with the untreated control.

The A-549 and MDA-MB-231 multicellular spheroids were treated to diverse concentrations between 100 and 800 *μ*g/mL of methanol and chloroform extracts, for 24, 48, and 72 h. The IC_50_ values of methanol and chloroform extracts on A-549 and MDA-MB-231 spheroids at 24, 48, and 72 h was shown in Table 4. The data in Figure 2 represented that the percentage of cell viability inhibition of the treated extracts on homogenous multicellular spheroids had become almost 4 to 5 times higher compared with the monolayer cells (2D), due to the lower sensitivity of solid tumor models to chemotherapeutics agents.

### 3.2. Morphological Evaluation Using Dual Acridine Orange/Ethidium Bromide Staining Assay

**Figure 3:**
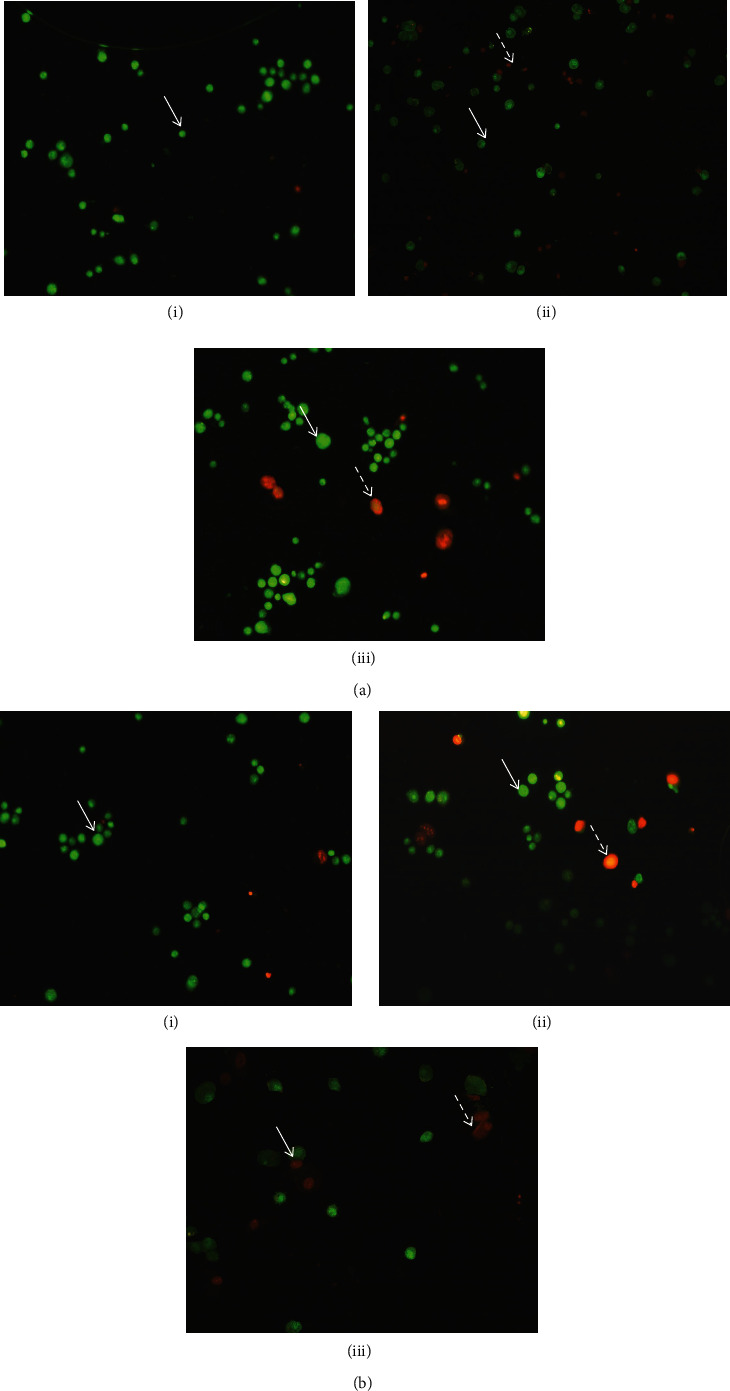
Dual acridine orange/ethidium bromide staining of A-549 (a) and MDA-MB-231 (b) cells with characteristic symptoms of apoptosis: (і) DMSO 0.1% as negative control and (іі) and (ііі) cells treated with IC_50_ values of etoposide, methanol (A-549), and chloroform (MDA-MB-231) extracts of *I. purpurea* leaves for 24 h. White arrow indicates live cells, and dashed arrow shows apoptotic cells. The images were taken with a fluorescence microscope at 40×.

Acridine orange emits green ﬂuorescence only in viable cells, although it penetrates both viable and dead cells, while ethidium bromide emits red ﬂuorescence in the necrotic and late stage of apoptotic cells. Our study revealed that the IC_50_ values of methanol and chloroform leaves extracts after 24 h, diminished cell viability, and promoted apoptosis in lung and breast cancer cells. As presented in Figure 3, the living cells appear green, while the apoptotic cells with orange particles in the nucleus are distinguished. The cells exposed to methanol and chloroform leaves extracts exhibited the expanded apoptosis relative to control and even etoposide treated cells.

### 3.3. Evaluation of Apoptosis by Flow Cytometry Analysis

**Figure 4:**
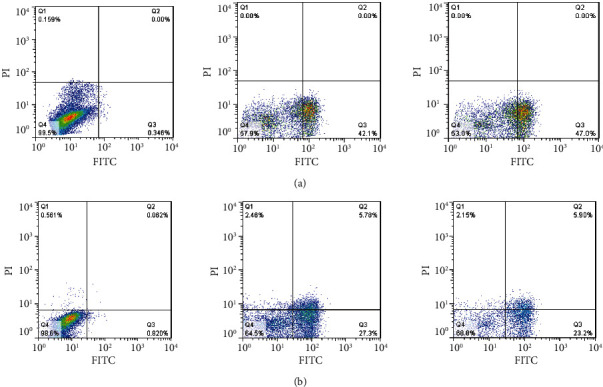
Flow cytometric analysis of A-549 (a) and MDA-MB-231 (b) cells treated with extracts of *I. purpurea* leaves. Cells were stained with Annexin V-FITC/PI and quantitated by flow cytometry after 24 h of incubation. The cells treated with DMSO 0.1% (negative control) or with IC_50_ value of etoposide (positive control), chloroform extract of *I. purpurea* leaves (MDA-MB-231), and methanol extract of *I. purpurea* leaves (A-549).

To explore whether methanol and chloroform leaves extracts showed cytotoxicity to A-549 and MDA-MB-231 cells through inducing apoptosis, the cells were stained with Annexin V-FITC/PI and then assessed by flow cytometry. Annexin V-FITC/PI staining followed by flow cytometry exhibited that cells involve apoptosis after exposure to IC_50_ values of methanol and chloroform leaves extracts. As represented in Figure 4, methanol and chloroform leaves extracts promoted apoptosis in lung and breast cancer cell lines. Our data provided that exposure of cells to IC_50_ values of methanol and chloroform extracts resulted in apoptosis of 47.0% early apoptosis in A-549 and 23.2% early and 5.9% late apoptosis in MDA-MB-231 cells after 24 h, respectively.

### 3.4. Inhibition of Cell Proliferation of A-549 Cells through Induction of Cell Cycle Arrest

**Figure 5:**
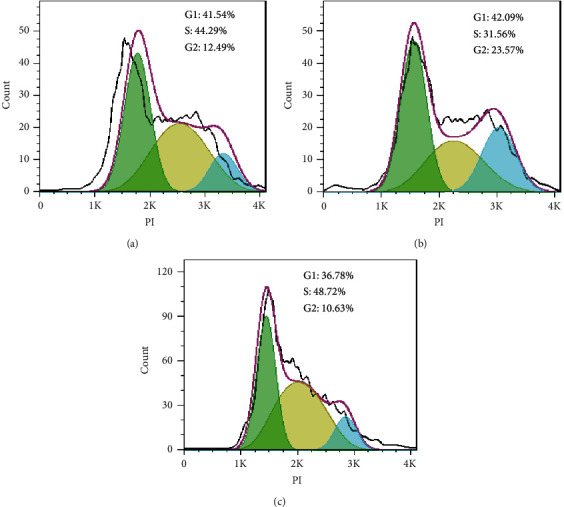
Flow cytometry cell cycle analysis of A-549 cells. Cell cycle analysis of A-549 cells treated with (a) DMSO 0.1% (negative control), (b) IC_50_ value of etoposide (positive control), and (c) methanol extract of *I. purpurea* leaves after 24 h.

To investigate the underlying mechanism of cell growth suppression, cell cycle analysis was performed on the A-549 cells exposed to the IC_50_ value of the methanol extract. The data in Figure 5(c) represented that exposure to the IC_50_ value of methanol extract slightly prohibited cell division of PI-stained A-549 cells after 24 h. Exposure to the IC_50_ value of this extract repressed DNA synthesis, and replication, which was exhibited by an increased percentage of S cells from 44.29% in the negative control cells to 48.72% in the extract-treated cells after 24 h (Figure 5). Overall, the percentage of S-phase cells gradually increased, while the number of cells in the *G*_0_/*G*_1_ and *G*_2_/M points were reduced. The ratio of cells in the S-phase increased distinctly in cells treated with this extract after 24 h, representing that methanol extract of *I. purpurea* leaves was promoted S-phase cell cycle arrest and apoptosis in A-549 lung cancer cells.

### 3.5. Caspase-3 Activation Assay

**Figure 6:**
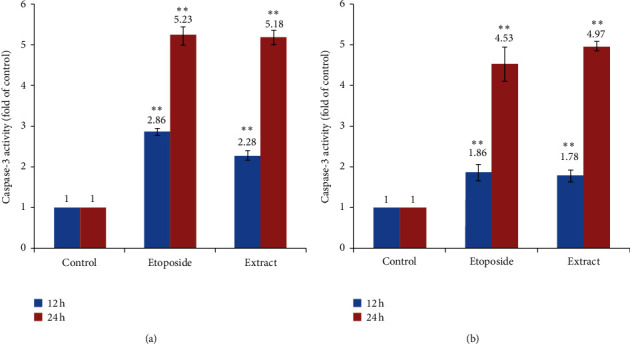
Caspase-3 activity in A-549 (a) and MDA-MB-231 (b) exposed to etoposide and extracts of *I. purpurea* leaves. The cells were treated with DMSO 0.1% (negative control), etoposide (positive control), and methanol and chloroform leaves extracts at the IC_50_ values for 12 and 24 h. The caspase-3 activity in the cell lysates was monitored by Ac-DEVD-pNA chromogenic substrate at 405 nm. Each value is the mean of three independent experiments ± SD. ^∗^Statistically significant differences between the treated cells compared with control according to Student's *t*-test (*P* < 0.01).

Caspase-3 is activated in the early stages of apoptosis, and as a critical effector protease, it plays a pivotal role in modulating apoptosis. In the finding of many potential anticancer agents, the activation of caspase-3 is very effective. Detection of active caspase-3 in A-549 and MDA-MB-231 cells for 12 and 24 h was investigated to observe whether the induction of cytotoxicity by IC_50_ values of methanol and chloroform leaves extracts is dependent on caspase-3 activity or not. Caspase-3 potency was expressed by altering activity in treated cells relative to control with a colorimetric assay. Our results revealed that the activity of these extracts for caspase-3 activation is similar to that of etoposide in both of the tested cell lines. A significant increase of approximately fivefold in caspase-3 activity was observed in A-549 and MDA-MB-231 cells exposed to IC_50_ values of methanol and chloroform leaves extracts relative to the control cells after 24 h with a significant difference of *P* < 0.01 (Figure 6).

### 3.6. Possible Apoptosis Pathways Caused by *I. purpurea* Extracts in A-549 Cells with Western Blotting

**Figure 7:**
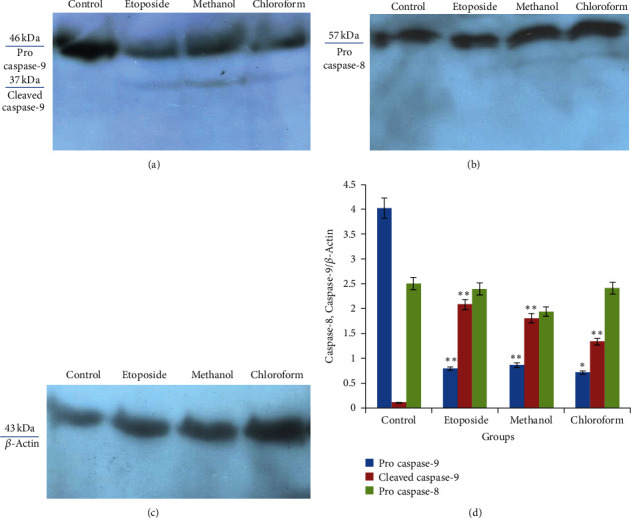
Cleavage of caspase-9 and caspase-8 processing in A-549 cell line upon exposure with DMSO 0.1% (negative control), IC_50_ values of etoposide (positive control) and methanol and chloroform extracts of *I. purpurea* for 24 h. Following treatment, the cells were lysed and analyzed by western blot with anti-caspase-9 and anti-caspase-8 antibodies. *β*-Actin (43 KD) was used to confirm equal protein loading. (a) The molecular weight of procaspase-9 and activated (cleaved) caspase-9 was 46 and 37 kD, respectively. (b) The molecular weight of procaspase-8 was 57 kD. (c) Semiquantitative analysis of western blot for caspase-9. The intensity of each band was quantified by ImageJ image processing program. All values were expressed as mean ± SD of at least three determinations. Significant differences are indicated by ^*∗*^*P* < 0.05 and ^*∗∗*^*P* < 0.01 as compared with the control group.

Caspase-3, an effector caspase, is activated by initiator caspases such as caspase-8 (in the extrinsic pathway of apoptosis) and caspase-9 (in the intrinsic way of apoptosis). To assess apoptosis-inducing, activation of caspase-8 and caspase-9 were determined by western blot assay using rabbit anti-caspase-8 and mouse anti-caspase-9 primary antibodies. The anti-caspase-9 antibody recognizes the pro-form and active form of this protease. The A-549 cells were treated to IC_50_ values of methanol and chloroform extracts of *I. purpurea* and etoposide (positive control) for 24 h. The finding from western blot provided that upon exposure to these extracts, the expression levels of procaspases-9 were significantly decreased, indicating procaspase cleavage and enzyme activation (Figure 7(a)). Analysis of western blotting of the cell lysates with an anti-caspase-9 antibody, which detected procaspase and cleaved fragments, showed caspase-9 activation with one cleaved form of caspase-9 with the molecular weight of 37 kDa. Considering that the molecular weight of procaspase-8 is 57 kDa and the weight of cleaved form is 42/43, we did not consider significant changes in procaspase band density of treated cells compared with control cells following 24 h treatment with extracts (Figure 7(b)).

## 4. Discussion

As an effective option, chemotherapy is currently used to treat many types of cancers alone or in combination with radiotherapy [9, 10, 20, 21]. Due to long-term sequelae and severe adverse effects of traditional chemotherapy, present studies focus on finding natural compounds that improve such side effects [5, 7, 13, 22–24]. Recently, plants have played a vital role in improving several clinically useful anticancer drugs, and it has been confirmed that over 60% of currently used antiproliferative compounds are directly or indirectly originated from natural sources [7, 25]. In traditional medical treatment, the role of herbal medicine is considerable; thus, recently, many studies have been directed to screen and evaluate the new plant-derived natural compounds [7, 25].

The current study reported the cytotoxic activities of aqueous and organic extracts of *I. purpurea* leaves to a set of human cancer cell lines, including A-549, HepG-2, and MDA-MB-231, and also elucidated the underlying molecular mechanisms. According to the results, most organic extracts showed major antiproliferative effects against tested human cancer cell lines, while there was no effect on the growth of MCF-10A as a normal breast cell. However, the water extract exhibited no acceptable activity at a concentration of 50 to 400 *µ*g/mL. Two extracts (methanol and chloroform) of *I. purpurea* showed the highest inhibition activity against the lung and breast cancer cell lines. Furthermore, these extracts suppressed the growth of lung and breast cancer cells in a concentration- and time-dependent manner. All of the extracts had lower cytotoxicity against MCF-10A cells compared with the cancer cells, suggesting that the extracts of *I. purpurea* leaves were selective towards cancer cells compared with those of normal. Importantly, no study, to the best of the authors' knowledge, has evaluated the anticancer activity of *I. purpurea* to date. However, a study conducted by Naz et al. on the species of *batatas* reveals the cytotoxic potential of this *Ipomoea* species on the MDBK bovine cancer cell line [26]. Correspondingly, with these studies, Zia-Ul-Haq et al. reported the antiproliferative effect of *I. hederacea* in lung and colon cancer [15]. In this regard, Fan et al. show the antiproliferative property of *I. aquatica* against human cancer cell lines, including MCF-7, SMMC-7721, HepG-2, MG-63, and U2-OS [26]. Moreover, the cytotoxic activity of *I. pes-caprae* is demonstrated in separate studies by Parekh and Ganjir research group [27, 28].

3D cell culture is capably bridging the gap between the traditional 2D cell culture model and *in vivo* whole animal system. It is well-documented that drug treatment response in 3D cell culture is more similar to human tumors compared with cells cultured as a monolayer on plastic [29–34]. The evidence of the antiproliferative activity assay on A-549 and MDA-MB-231 spheroids showed that the IC_50_ values of methanol and chloroform extracts on spheroids was almost 4 to 5 times higher than that of 2D cell cultures. Thus, the current research indicates that spheroids in 3D culture were more resistant to the toxic properties of the most potent extract in comparison with conventional monolayer cells. Spheroids are believed to mimic the natural tumor behaviour and microenvironment more effectively than corresponding 2D cultures [35, 36].

It is well-documented that most chemotherapeutic drugs induce cell death with morphological features of apoptosis in cancer cells. Therefore, apoptosis induction by chemotherapeutic agents can be a physiological advantage for cancer treatment [3, 21, 22]. Of all, methanol and chloroform extracts showed the highest cytotoxic potency; therefore, the same extracts are used for apoptotic determination. Further examinations, including dual acridine orange/ethidium bromide and Annexin V-FITC/PI staining, flow cytometric analysis, cell cycle analysis, caspase-3 colorimetric assay, and initiator caspase immunoblotting were indicated that the selected extracts promote apoptosis through the activation of caspase-9 and caspase-3 in lung and breast cancer cell lines. The finding from dual acridine orange/ethidium bromide staining exhibited that the IC_50_ values of methanol and chloroform leaves extracts declined cell viability and promoted apoptosis in A-549 and MDA-MB-231 cancer cell lines. Annexin V-FITC/PI staining exhibited that the methanol and chloroform extract promoted early apoptosis in the A-549 and also early and late apoptosis in MDA-MB-231 cells after 24 h of incubation.

In this study, the cell cycle arrest, as a mechanism of natural cytotoxic compounds, was evaluated since some natural compounds inhibit the proliferation of cancer cells by suppressing the cell cycle. The effect of the most potent extract (methanol) of *I. purpurea* leaves on the cell-cycle progress of A-549cells was assessed by flow cytometry. According to recent studies, one of the induced cell death mechanisms of medicinal plant-derived extracts could be arresting *G*_0_/*G*_1_, S, or *G*_2_/M phases, promoting apoptosis. Following the exposure of A-549 cells with the IC_50_ value of the methanol extract of *I. purpurea*, findings confirmed this extract's potential to arrest A-549 lung cancer cells at S-phase considerably. Furthermore, it was shown that methanol extract of *I. purpurea* leaves by blocking DNA synthesis leads to S-phase cell cycle arrest and proliferation inhibition in A-549 cells. In this regard, Fan et al. reported that *I. aquatica* inhibits growth and promotes the *G*_0_/*G*_1_-phase cell cycle arrest and the apoptosis induction in HepG-2 cancer cells [26].

Caspase-3, as the main executioner and key effector activated in both pathways involved in triggering apoptosis, includes intrinsic and extrinsic pathways, affecting the drug cytotoxicity. Colorimetric caspase-3 activity assay in the cells exhibited *I. purpurea* leaves extracts' ability to activate caspase-3 in a time-dependent manner. The comparison of caspase-3 activity in treated cells with negative control cells indicated a significant increase in caspase-3 activity in A-549 exposed cells to the methanol and in MDA-MB-231 cells exposed to chloroform leaves extracts (IC_50_) about five-fold (*P* < 0.01). This study revealed that *I. purpurea* leaves extracts promote apoptosis induction through a caspase-3 dependent way in cancer cells.

To confirm whether the intrinsic or the extrinsic apoptosis pathway is involved in the toxicity, the processing of the caspase-8 and caspase-9, as initiator caspases of the extrinsic and intrinsic paths, is examined by the western blot assay. The immunoblotting assay of the cell lysates with anti-caspase-9 and caspase-8 antibodies revealed caspase-9 activation following 24 h treatment of the A-549 cells with the *I. purpurea* leaves extracts. Furthermore, the present research results have shown that *I. purpurea* leaves extracts to induce the activation of caspase-9, as one of the key markers of the activation of the apoptosis mitochondrial or intrinsic pathway. Similar to the current study, Sugata et al. reported that *I. batatas* could promote apoptosis induction in MCF-7 cancer cells through the extrinsic and intrinsic pathways and in SNU-1 cancer cells through the caspase-3 activation [36]. Some antiproliferative agents initiate cell death through the extrinsic pathway through death receptors on the surface of the cell membrane; still, most research explains that anticancer agents induce apoptosis through the intrinsic mitochondrial pathway. The *I. purpurea* leaves extract promoted the S-phase cell cycle arrest and apoptosis in A-549 lung cancer cells through the mitochondrial pathway. Furthermore, the present work demonstrated that *I. purpurea* leaves chloroform extract had an inhibitory effect on MDA-MB-231 breast cancer cells *in vitro*. Moreover, these extracts promoted apoptosis induction through the caspase-dependent mitochondrial pathway and cell cycle arrest.

## 5. Conclusion

The results suggested that *I. purpurea* leaves extracts inhibited the proliferation of human lung and breast cancer (A-549 and MDA-MB-231) cells. Conclusively, the *I. purpurea* methanol and chloroform extracts indicated the ability to activate potent anticancer, promote cell cycle arrest of A-549 cells in the S-phase, and induce apoptosis in lung and breast cancer cell lines through a caspase-dependent pathway. The result of the present study could be helpful for further examination, aiming at chemically identifying the specific compounds responsible for the antiproliferative properties of *I. purpurea* methanol and chloroform extracts. Finally, the present research also revealed that the potential antiproliferative effect of *I. purpurea* leaves against lung cancer A-549 cells involved mitochondrially mediated apoptosis, preparing evidence for further study.

## Figures and Tables

**Table 1:** *In vitro* the antiproliferative activity (IC_50_) of extracts of *I. purpurea* leaves after 24 h of treatment.

IC_50_ (*μ*g/ml)^*∗*^				
Extracts	A-549	MDA-MB-231	HepG-2	MCF-10A
Chloroform	198.66 ± 0.96	235.50 ± 2.15	>400.0	<5,000.0
Methanol	196.37 ± 1.29	>400.0	289.68 ± 3.78	<5,000.0
Hexane	>400.0	>400.0	>400.0	<5,000.0
Ethyl acetate	>400.0	>400.0	>400.0	<5,000.0
Aqueous	>400.0	>400.0	>400.0	<5,000.0
Etoposide	29.21 ± 0.19	35.91 ± 0.82	27.26 ± 1.09	129.31 ± 2.07

^*∗*^Values are means of three replicate samples (*n* = 3). Data are presented as the mean ± SEM. Results were analyzed using descriptive statistics.

**Table 2:** *In vitro* the antiproliferative activity (IC_50_) of extracts of *I. purpurea* leaves after 48 h of treatment.

IC_50_ (*μ*g/ml)^*∗*^				
Extracts	A-549	MDA-MB-231	HepG-2	MCF-10A
Chloroform	157.41 ± 6.93	124.54 ± 0.01	207.73 ± 2.45	<5,000.0
Methanol	53.62 ± 0.07	224.11 ± 2.28	157.50 ± 0.13	<5,000.0
Hexane	>400.0	>400.0	>400.0	<5,000.0
Ethyl acetate	160.46 ± 6.30	135.67 ± 0.00	>400.0	<5,000.0
Aqueous	>400.0	>400.0	>400.0	<5,000.0
Etoposide	16.58 ± 0.78	20.30 ± 0.21	17.40 ± 0.49	96.31 ± 1.31

^*∗*^Values are means of three replicate samples (*n* = 3). Data are presented as the mean ± SEM. Results were analyzed using descriptive statistics.

**Table 3:** *In vitro* the antiproliferative activity (IC_50_) of extracts of *I. purpurea* leaves after 72 h of treatment.

IC_50_ (*μ*g/ml)^*∗*^				
Extracts	A-549	MDA-MB-231	HepG-2	MCF-10A
Chloroform	137.03 ± 2.6	119.24 ± 3.4	190.02 ± 3.4	<5,000.0
Methanol	48.35 ± 3.09	198.19 ± 6.4	134.91 ± 2.7	<5,000.0
Hexane	>400.0	>400.0	>400.0	<5,000.0
Ethyl acetate	157.29 ± 4.6	119.53 ± 1.6	>400.0	<5,000.0
Aqueous	>400.0	>400.0	>400.0	<5,000.0
Etoposide	13.11 ± 1.04	18.40 ± 0.68	15.22 ± 1.34	85.62 ± 2.02

^*∗*^Values are means of three replicate samples (*n* = 3). Data are presented as the mean ± SEM. Results were analyzed using descriptive statistics.

**Table 4:** *In vitro* the antiproliferative activity (IC_50_) of chloroform and methanol extracts of *I. purpurea* leaves on 3D spheroids of MDA-MB-231 and A-549, respectively, after 24, 48, and 72 h of treatment.

IC_50_ (*μ*g/ml)^*∗*^			
Extracts	24 hours	48 hours	72 hours
Chloroform	>800.0	478.35 ± 1.93	487.25 ± 1.34
Methanol	>800.0	275.18 ± 3.02	255.19 ± 1.78
Etoposide	587.04 ± 4.78	201.85 ± 2.26	149.50 ± 2.78

^*∗*^Values are means of three replicate samples (*n* = 3). Data are presented as the mean ± SEM. Results were analyzed using descriptive statistics.

## Data Availability

The data will be made available upon request.

## Supplementary Materials

Supplementary Materials The cell viability inhibition of all cancer cells three times (24, 48, and 72 h) was presented in Supplementary Information (Figures S1, S2, and S3).

## References

[1] Charoensin S. (2014). Antioxidant and anticancer activities of *Moringa oleifera* leaves. *Journal of Medicinal Plants Research*.

[2] Arome D., Amarachi A. (2014). A review on herbal plants with anti-tumour properties. *Journal of Pharmaceutical, Chemical and Biological Sciences*.

[3] Rahmani-Nezhad S., Safavi M., Pordeli M. (2014). Synthesis, in vitro cytotoxicity and apoptosis inducing study of 2-aryl-3-nitro-2H-chromene derivatives as potent anti-breast cancer agents. *European Journal of Medicinal Chemistry*.

[4] Prakash O., Kumar A., Kumar P., Ajeet A. (2013). Anticancer potential of plants and natural products: a review. *American Journal of Pharmacological Sciences*.

[5] Safavi M., Esmati N., Ardestani S. K. (2012). Halogenated flavanones as potential apoptosis-inducing agents: synthesis and biological activity evaluation. *European Journal of Medicinal Chemistry*.

[6] Adedapo A. A., Oyagbemi A. A., Fagbohun O. A., Omobowale T. O., Yakubu M. A. (2016). Evaluation of the anticancer properties of the methanol leaf extract of *Chromolaena odorata* on HT-29 cell line. *Journal of Pharmacognosy and Phytochemistry*.

[7] El-Naggar S. A., Abdel-Farid I. B., Elgebaly H. A., Germoush M. O. (2015). Metabolomic profiling, antioxidant capacity and *in vitro* anticancer activity of some compositae plants growing in Saudi Arabia. *African Journal of Pharmacy and Pharmacology*.

[8] Zhu G., Conner S. E., Zhou X. (2003). Synthesis, Structure−Activity relationship, and biological studies of indolocarbazoles as potent cyclin D1-CDK4 inhibitors. *Journal of Medicinal Chemistry*.

[9] Zhuo Z., Hu J., Yang X. (2015). Ailanthone inhibits Huh7 cancer cell growth via cell cycle arrest and apoptosis *in vitro* and *in vivo*. *Scientific Reports*.

[10] Behzad, Pirani A., Mosaddegh M. (2014). Cytotoxic activity of some medicinal plants from Hamedan district of Iran. *Iranian Journal of Pharmaceutical Research: IJPR*.

[11] Cragg G. M., Newman D. J. (2005). Plants as a source of anti-cancer agents. *Journal of Ethnopharmacology*.

[12] Uddin S. J., Grice I. D., Tiralongo E. (2011). Cytotoxic effects of Bangladeshi medicinal plant extracts. *Evidence-Based Complementary and Alternative Medicine*.

[13] Namian P., Talebi T., Ghasemi Germi K., Shabani F. (2013). Screening of biological activities (antioxidant, antibacterial and antitumor) of *Nerium oleander* leaf and flower extracts. *American Journal of Phytomedicine and Clinical Therapeutics*.

[14] Thingujam D., Rekha K., Megala J., Usha B. (2015). Antioxidant and anticancer properties of Catharanthus pusillus. *International Journal of Advance and Innovative Research*.

[15] Zia-Ul-Haq M., Riaz M., Feo V. (2012). *Ipomea hederacea* Jacq.: a medicinal herb with promising health benefits. *Molecules*.

[16] Meira M., Silva E. P. D., David J. M., David J. P. (2012). Review of the genus *Ipomoea*: traditional uses, chemistry and biological activities. *Revista Brasileira de Farmacognosia*.

[17] Srivastava D. (2017). Medicinal plant of genus *ipomoea*: present scenario, challenges and future, prospective. *Research Journal of Recent Sciences*.

[18] Abolhasani M. H., Safavi M., Goodarzi M. T., Kassaee S. M., Azin M. (2018). Identification and anti-cancer activity in 2D and 3D cell culture evaluation of an Iranian isolated marine microalgae *Picochlorum* sp. RCC486. *DARU Journal of Pharmaceutical Sciences*.

[19] Jamali, Kavoosi G., Safavi M., Ardestani S. K. (2018). *In-vitro* evaluation of apoptotic effect of OEO and thymol in 2D and 3D cell cultures and the study of their interaction mode with DNA. *Scientific Reports*.

[20] Yaacob N. S., Hamzah N., Nik Mohamed Kamal N. N. (2010). Anticancer activity of a sub-fraction of dichloromethane extract of *Strobilanthes crispus* on human breast and prostate cancer cells *in vitro*. *BMC Complementary and Alternative Medicine*.

[21] Gavamukulya Y., Abou-Elella F., Wamunyokoli F., AEl-Shemy H. (2014). Phytochemical screening, anti-oxidant activity and in vitro anticancer potential of ethanolic and water leaves extracts of *Annona muricata* (Graviola). *Asian Pacific Journal of Ttropical Medicine*.

[22] Oskoueian E., Abdullah N., Saad W. Z. (2011). Antioxidant, anti-inflammatory and anticancer activities of methanolic extracts from *Jatropha curcas* Linn. *Journal of Medicinal Plants Research*.

[23] Jung (2014). Soluble extract from *Moringa oleifera* leaves with a new anticancer activity. *PLos One*.

[24] Ghagane S. C., Puranik S. I., Kumbar V. M. (2017). *In vitro* antioxidant and anticancer activity of *Leea indica* leaf extracts on human prostate cancer cell lines. *Integrative Medicine Research*.

[25] Naz S., Naqvi S. A. R., Khan Z. A. (2017). Antioxidant, antimicrobial and antiproliferative activities of peel and pulp extracts of red and white varieties of *Ipomoea batatas* (L) Lam. *Tropical Journal of Pharmaceutical Research*.

[26] Fan B.-Y., Gu Y.-C., He Y., Li Z.-R., Luo J.-G., Kong L.-Y. (2014). Cytotoxic resin glycosides from Ipomoea aquatica and their effects on intracellular Ca2+Concentrations. *Journal of Natural Products*.

[27] Parekh K., Patel A., Modi A., Chandrashekhar H. R. (2012). Antioxidant and cytotoxic activities of few selected *Ipomoea* species. *Pharmacologia*.

[28] Ganjir M., Behera D. R., Bhatnagar S. (2013). Phytochemical analysis, cytotoxic and antioxidant potential of *Ipomoea pes caprae* (L) R. Br and *merremia umbellata* (L.) H. Hallier. *International Journal of Scientific & Technology Research*.

[29] Joseph J. S., Malindisa S. T., Ntwasa M. (2018). Two-dimensional (2D) and three-dimensional (3D) cell culturing in drug discovery. *Cell Culture*.

[30] Lee, Lilly G. D., Doty R. C., Podsiadlo P., Kotov N. A. (2009). *In vitro* toxicity testing of nanoparticles in 3D cell culture. *Small (Weinheim an der Bergstrasse, Germany)*.

[31] Melissaridou S., Wiechec E., Magan M. (2019). The effect of 2D and 3D cell cultures on treatment response, EMT profile and stem cell features in head and neck cancer. *Cancer Cell International*.

[32] Kunz-Schughart L. A., Freyer J. P., Hofstaedter F., Ebner R. (2004). The use of 3-D cultures for high-throughput screening: the multicellular spheroid model. *Journal of Biomolecular Screening*.

[33] Sirenko O., Mitlo T., Hesley J., Luke S., Owens W., Cromwell E. F. (2015). High-content assays for characterizing the viability and morphology of 3D cancer spheroid cultures. *Assay and Drug Development Technologies*.

[34] Salehi F., Behboudi H., Kavoosi G., Ardestani S. K. (2017). Monitoring ZEO apoptotic potential in 2D and 3D cell cultures and associated spectroscopic evidence on mode of interaction with DNA. *Scientific Reports*.

[35] Galateanu B., Hudita A., Negrei C. (2016). Impact of multicellular tumor spheroids as an in vivo-like tumor model on anticancer drug response. *International Journal of Oncology*.

[36] Sugata M., Lin C.-Y., Shih Y.-C. (2015). Anti-inflammatory and anticancer activities of taiwanese purple-fleshed sweet potatoes (*Ipomoea batatas* L. Lam) extracts. *BioMed Research International*.

