# Supplementary material for: Anticancer Activity of Ipomoea purpurea Leaves Extracts in Monolayer and Three-Dimensional Cell Culture
Source: Evid Based Complement Alternat Med. 2021 Jun 7;2021:6666567. doi: 10.1155/2021/6666567 (PMC8205580; doi:10.1155/2021/6666567)
Supplement: Supplementary Materials — The cell viability inhibition of all cancer cells three times (24, 48, and 72 h) was presented in Supplementary Information (Figures S1, S2, and S3). [file 6666567.f1.docx]

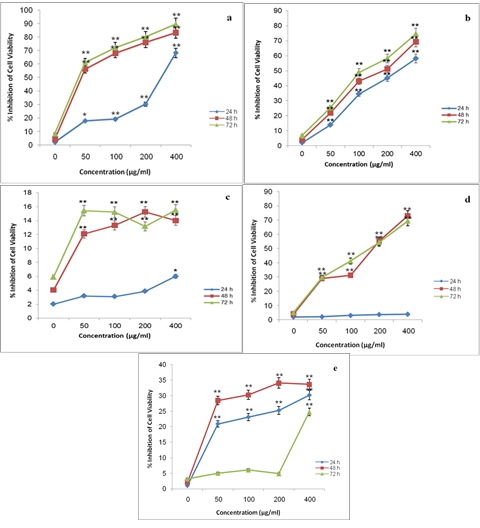


Figure S1: The Cell viability inhibition of A-549 cell line treated with different concentrations of methanol (a), chloroform (b), hexane (c), ethyl-acetate (d) and aqueous (e) extracts of *I. purpurea* for 24, 48 and 72 h was determined using the MTT assay. The data are expressed as the mean ± SD of three independent experiments. Significant differences are indicated by **P*< 0.05 and ***P*< 0.01 as compared with the untreated control.


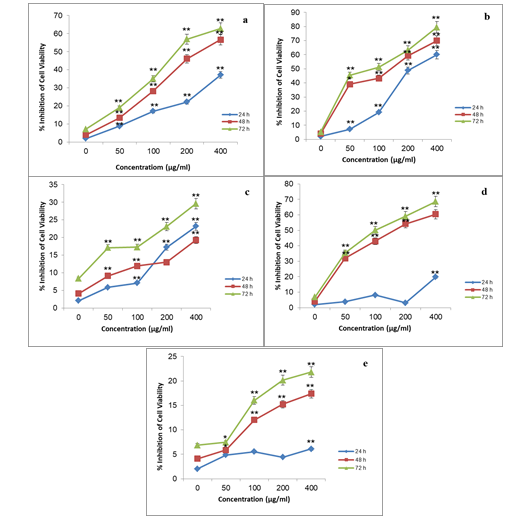


Figure S2: The Cell viability inhibition of MDA-MB-231 cell line treated with different concentrations of methanol (a), chloroform (b), hexane (c), ethyl-acetate (d) and aqueous (e) extracts of *I. purpurea* for 24, 48 and 72 h was determined using the MTT assay. The data are expressed as the mean ± SD of three independent experiments. Significant differences are indicated by **P*< 0.05 and ***P*< 0.01 as compared with the untreated control.


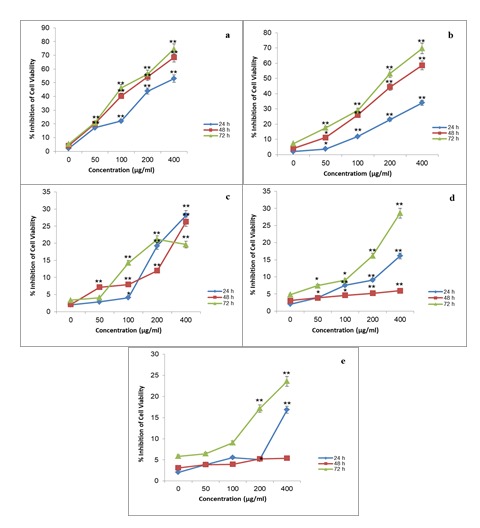


Figure S3: The Cell viability inhibition of HepG2 cell line treated with different concentrations of methanol (a), chloroform (b), hexane (c), ethyl-acetate (d) and aqueous (e) extracts of *I. purpurea* for 24, 48 and 72 h was determined using the MTT assay. The data are expressed as the mean ± SD of three independent experiments. Significant differences are indicated by **P*< 0.05 and ***P*< 0.01 as compared with the untreated control.
